# Beyond Remodeling: Four Structural–Functional Recovery Patterns as a Framework for Interpreting Ventricular Recovery After Reperfused STEMI

**DOI:** 10.3390/biomedicines14102185

**Published:** 2026-09-27

**Authors:** Alina Crăciun-Mirescu, Oana Mirea, Despina Emanuela Toader, Mihaela-Corina Berceanu, Virginia Maria Rădulescu, Cristiana Gianina Moise, Roxana-Daniela Delcea, Constantin Militaru, Cristian Militaru

**Affiliations:** 1 Doctoral School, University of Medicine and Pharmacy of Craiova, 200349 Craiova, Romania; alinameds@yahoo.com (A.C.-M.); stroescu_roxana@yahoo.com (R.-D.D.); 2 Department of Cardiology, Craiova Emergency Clinical County Hospital, 200642 Craiova, Romania; despinamtoader@yahoo.com (D.E.T.); cristiana.moise10@gmail.com (C.G.M.); cccmilitaru@yahoo.com (C.M.); crmilitaru@gmail.com (C.M.); 3 Department of Cardiology, University of Medicine and Pharmacy of Craiova, 200349 Craiova, Romania; mihaela.berceanu@umfcv.ro; 4 Department of Cardiovascular Surgery, Craiova Emergency Clinical County Hospital, 200642 Craiova, Romania; 5 Department of Medical Informatics and Biostatistics, University of Medicine and Pharmacy of Craiova, 200349 Craiova, Romania; virginia.radulescu@umfcv.ro; 6 Department of Physiology, University of Medicine and Pharmacy of Craiova, 200349 Craiova, Romania

**Keywords:** echocardiography, three-dimensional, global longitudinal strain, left atrial strain, myocardial work, ST-elevation myocardial infarction, ventricular remodeling

## Abstract

**Background/Objectives**: Ventricular recovery after ST-segment elevation myocardial infarction (STEMI) is commonly assessed using either structural (volumetric) or functional (ejection-fraction) criteria, although these may capture different aspects of recovery. We examined whether their cross-classification identifies distinct recovery patterns and whether myocardial mechanics and left atrial function provide additional information. **Methods**: We prospectively followed 101 patients with first STEMI and single-vessel coronary artery disease treated with PCI. Echocardiography was performed 12–24 h after reperfusion and at 12 ± 2 months. Structural recovery (≥15% reduction in 3D LV end-systolic volume) and functional recovery (≥5-point absolute LVEF increase) were cross-classified into four recovery patterns. **Results**: Among 53 patients with complete paired measurements, 10 (18.9%) showed concordant recovery, 7 (13.2%) isolated structural recovery, 9 (17.0%) isolated functional recovery, and 27 (50.9%) no recovery; 30.2% therefore showed discordant recovery. The no-recovery group was heterogeneous, including both volumetric deterioration and stable or improving volumes below the recovery threshold. Continuous changes in 3D-ESV and LVEF were only moderately correlated (ρ = 0.353, *p* = 0.0095), and discordance persisted in sensitivity analyses. Myocardial mechanics and LASR did not differentiate recovery patterns, but several of these baseline markers showed exploratory associations with clinical outcomes after false-discovery-rate correction. **Conclusions**: Structural and functional recovery after STEMI are complementary rather than interchangeable, and this discordance persisted across extensive sensitivity analyses; however, assignment of individual patients to specific recovery categories remained sensitive to the chosen thresholds and LVEF measurement method. Myocardial mechanics and LASR may provide additional information about clinical expression.

## 1. Introduction

Following myocardial revascularization and guideline-directed medical therapy (GDMT), post-infarction myocardial healing encompasses inflammation and edema resolution, recovery from stunning, and scar maturation. This is clinically expressed through ventricular remodeling [1], a heterogeneous process that may evolve toward reverse or adverse remodeling, influencing long-term ventricular function and clinical outcomes [2].

Beyond infarct size and reperfusion, the biological milieu of acute STEMI may also influence subsequent myocardial injury and recovery. In hyperglycemic STEMI, intracoronary thrombi have been reported to exhibit greater thrombus burden and enhanced inflammatory and oxidative activity, features associated with adverse clinical outcomes [3]. Microbial colonization of coronary thrombi has also been proposed as a contributor to atherothrombotic burden in this setting [4].

Ventricular remodeling is commonly assessed through left ventricular volumes and ejection fraction (LVEF).

In current practice, volumetric and LVEF-based assessments are often treated as interchangeable, although they are only moderately correlated: volumetric reverse remodeling can occur without EF increase, and LVEF may improve without measurable volume reduction [5]. Both describe chamber-level recovery and are influenced by ventricular geometry, loading conditions, and myocardial mechanics; neither directly measures myocardial healing [6].

Whether these two parameters evolve concordantly within individual patients has direct implications for how post-infarction recovery is defined and interpreted. Reverse remodeling is conventionally assessed as a binary outcome at a predefined follow-up time point, treating recovery as a completed event. Yet patients failing conventional recovery thresholds are unlikely to be homogeneous, as different structural and functional patterns may converge below the same endpoint [7,8]. Previous three-dimensional echocardiographic studies classified remodeling using structural categories alone, whereas integrating LV dilatation with the direction of LVEF change refined prognostic stratification but remained focused on adverse remodeling and did not define functional recovery using an absolute LVEF threshold [9,10]. Building on this work, the present study examines structural and functional recovery, rather than adverse remodeling, as complementary parameters, further characterized through myocardial deformation and left atrial function. It remains unclear how often structural and functional recovery occur in the same patient after STEMI and what is missed when either is considered alone.

The primary aim of this study was to cross-classify separately defined structural and functional recovery criteria, combining a reduction in three-dimensional end-systolic volume with an absolute LVEF increase to distinguish concordant, discordant, and no-recovery patterns. As a secondary aim, we examined whether echocardiographic markers (myocardial deformation, work indices, and left atrial strain) relate to pattern membership and to clinical status at follow-up, testing whether these measures provide information beyond chamber-level classification alone.

## 2. Materials and Methods

### 2.1. Study Design and Population

We prospectively enrolled 101 consecutive patients with a first ST-segment elevation myocardial infarction (STEMI) treated with percutaneous coronary intervention (PCI) between 19 September 2024 and 7 August 2025. All participants were therefore enrolled after ethics approval had been obtained on 18 September 2024. All patients underwent a comprehensive baseline echocardiographic examination 12–24 h after PCI, including two- and three-dimensional ventricular quantification, myocardial deformation imaging, myocardial work analysis, and left atrial strain assessment. Patients were scheduled for a protocol follow-up visit at 12 ± 2 months, with clinical, electrocardiographic, and echocardiographic reassessment.

The inclusion criteria were as follows: (1) consecutive patients presenting with a first STEMI; (2) presentation within 24 h of symptom onset; (3) angiographically confirmed single-vessel coronary artery disease; and (4) treatment with culprit-vessel PCI, with or without pre-PCI thrombolysis.

All patients underwent culprit-vessel PCI according to standard institutional protocol. Guideline-directed medical therapy for coronary syndrome, heart failure, secondary cardiovascular prevention, and comorbidities at follow-up (antiplatelet agents, beta-blockers, renin-angiotensin-system inhibitors [ACE inhibitor, ARB, or ARNI], SGLT2 inhibitors, mineralocorticoid receptor antagonists, statins, and diuretics) was recorded for the reassessed cohort.

The exclusion criteria were as follows: prior known cardiomyopathy; congenital heart disease; significant primary valvular heart disease (moderate or greater stenosis or regurgitation); other non-ischemic structural heart disease; multivessel coronary artery disease; and poor acoustic window precluding analysis. The original study protocol additionally listed inability to complete follow-up evaluation as an exclusion criterion; on review, this was not applied as a distinct prospective exclusion in practice, and all 101 enrolled patients were considered eligible for follow-up at enrollment. Of the 43 patients who did not complete the protocol follow-up visit, 42 were lost to follow-up during the study period and one died before the scheduled reassessment could take place (Section 3.6).

### 2.2. Echocardiographic Assessment

Examinations used a Vivid E95 system (GE HealthCare, Horten, Norway) with a 4V-D matrix-array transducer. Three-dimensional full-volume LV datasets used multi-beat, or single-beat when infeasible, stitched acquisition. LV volumes and ejection fraction were quantified using 4D Auto LVQ (EchoPAC v204, GE HealthCare), with manual adjustment when needed. Two-dimensional speckle-tracking—global longitudinal strain (GLS), myocardial work indices (global work index [GWI], global constructive work [GCW], global wasted work [GWW], global work efficiency [GWE]), peak strain dispersion (PSD), and left atrial strain (LASR, LAScd, LASct)—used Automated Function Imaging (AFI LV, AFI LA) in **EchoPAC v204** (GE HealthCare, Horten, Norway). GLS averaged peak systolic longitudinal strain across apical four-, two-, and three-chamber views; myocardial work indices used the pressure–strain loop method [11], with LV pressure non-invasively estimated from brachial cuff pressure. Left atrial strain used QRS onset as zero-reference [12]. Acquisition and analysis were performed at our EACVI-accredited laboratory (SCJU Craiova, Romania), per current EACVI/ASE recommendations [13,14], by a single experienced observer, who analyzed follow-up studies blinded to the corresponding baseline measurements. Intraobserver and interobserver reproducibility for the two threshold-defining variables were subsequently assessed in a random patient subset (Section 2.6).

### 2.3. Definitions of Structural and Functional Recovery

Structural recovery was defined as a ≥15% reduction in 3D end-systolic volume (3D-ESV) from baseline to follow-up, consistent with previously used volume-based reverse-remodeling thresholds after myocardial infarction, whether defined via end-diastolic volume [15] or, as applied here, end-systolic volume [16]. Functional recovery was defined as a ≥5-percentage-point absolute increase in biplane Simpson LVEF (two-dimensional). This magnitude corresponds to the lower bound of the 5- to 10-percentage-point range used across the literature to define LVEF-based functional recovery [16], and biplane 2D LVEF was chosen because it is acquired separately from the 3D dataset used for structural recovery, thereby reducing shared acquisition-related measurement error between the two criteria. These criteria were assessed separately and cross-classified to define four structural–functional recovery patterns: concordant recovery (both criteria fulfilled), isolated structural recovery (structural criterion only), isolated functional recovery (functional criterion only), and no recovery (neither criterion fulfilled). For the purposes of this study, “recovery pattern” refers to a structural–functional recovery category predefined for the present analysis rather than a data-driven or biologically validated pattern. Pattern assignment was restricted to patients with complete paired 3D-ESV and LVEF measurements. Sensitivity analyses included alternative thresholds for 3D-ESV reduction (≥10% and ≥20%), a more conservative functional-recovery threshold (≥10-point absolute LVEF increase) [17], continuous threshold-free analysis, and reclassification using 3D- and AFI-derived LVEF.

As a post hoc descriptive analysis, patients meeting neither recovery criterion were further stratified according to baseline LVEF (<50% vs. ≥50%) and the direction of 3D-ESV change (increase vs. no increase). This sub-stratification was intended to explore heterogeneity within the no-recovery pattern and was not considered an independently validated classification.

### 2.4. Clinical Outcomes

As an exploratory, hypothesis-generating analysis, we examined whether pattern membership and baseline echocardiographic parameters were associated with clinical status at follow-up, including NYHA class ≥ II, an exploratory HF-related clinical-status outcome, cardiovascular-cause rehospitalization, and a composite adverse-event endpoint (the HF-related clinical-status outcome, cardiovascular-cause rehospitalization, implantable cardioverter-defibrillator or cardiac resynchronization therapy implantation, major adverse cardiac events [recurrent myocardial infarction, stroke, cardiovascular death, or unplanned revascularization], or death). Cardiovascular-cause rehospitalization was ascertained from the discharge diagnosis documented during routine clinical care, rather than through a separate blinded study-specific adjudication process. The HF-related clinical-status outcome was defined as NYHA class ≥ II at reassessment or a documented clinical diagnosis of heart failure made by a treating physician at index-hospital discharge, during a subsequent hospitalization, or in the outpatient setting at any time between the index STEMI and follow-up; at index-hospital discharge, this diagnosis reflected clinical manifestations of HF documented during the acute hospitalization. This study-specific outcome was intended to capture symptomatic or clinically recognized HF burden and was not an adjudicated heart-failure event according to standardized clinical-trial endpoint definitions. For the marker–outcome screening analysis predefined for the present analysis, the three outcomes were NYHA class ≥ II, the HF-related clinical-status outcome, and the composite endpoint. Clinical outcome ascertainment was performed independently of, and blinded to, echocardiographic findings.

### 2.5. Statistical Analysis

Continuous variables are reported as mean ± SD or median [IQR], and categorical variables as counts (%). Differences in baseline continuous variables across the four recovery patterns were assessed using the Kruskal–Wallis test; paired baseline-to-follow-up changes using the Wilcoxon signed-rank test; categorical variables using the chi-squared or Fisher’s exact test; and associations between continuous variables using Spearman’s rank correlation. For binary clinical outcomes, univariable logistic regression was used to estimate odds ratios (OR) per 1-SD increase in standardized predictors, with discrimination summarized by AUC and incremental model fit assessed using likelihood-ratio tests of nested models. Given the exploratory nature, nominal *p*-values are reported alongside false-discovery-rate-adjusted results, where applicable, and should be interpreted as hypothesis-generating. For exploratory clinical-outcome analyses, Benjamini–Hochberg false-discovery-rate correction was applied across the family of 21 comparisons predefined for the present analysis, comprising seven echocardiographic markers (GLS, GWI, GCW, GWW, GWE, PSD, and LASR) and three clinical outcomes. Two further families, similarly predefined for the present analysis, used the same procedure: baseline echocardiographic comparisons across recovery patterns (LVEF, GLS, GWI, GCW, GWE, and LASR; Section 3.3) and baseline comparisons between reassessed and non-reassessed patients (age, sex, LVEF, GLS, and infarct territory; Section 4.6). Analyses were complete-case, with denominators reported in text or tables; two-sided *p* < 0.05 was considered nominally significant. Agreement between LVEF measurement methods was quantified using Cohen’s κ and Spearman correlation (Supplementary Table S1). Bootstrap resampling was used to derive 95% confidence intervals for the Spearman correlation and LVEF-method agreement (2000 iterations) and for AUC estimates (10,000 stratified iterations); optimism-corrected AUC estimates used 2000 bootstrap refits. Agreement between the structural and functional recovery criteria themselves was quantified using Cohen’s κ with a bootstrap 95% confidence interval, the observed agreement, and an exact McNemar test for asymmetry of disagreement; the discordance proportion is reported with an exact binomial 95% confidence interval. Robustness of this agreement was examined across a grid of six structural (≥5% to ≥30% 3D-ESV reduction) and seven functional (≥2 to ≥10 LVEF points) thresholds, with both recovery criteria derived from the same three-dimensional dataset, with a relative functional criterion (≥10% relative LVEF increase), and within strata of baseline LVEF (<50% vs. ≥50%). Data were collected and managed in Microsoft Excel 2021 (Microsoft Corporation, Redmond, WA, USA). Standard statistical tests were performed using IBM SPSS Statistics, version 26.0 (IBM Corp., Armonk, NY, USA); Firth penalized regression, bootstrap resampling, and the exact conditional and threshold-grid analyses were performed in Python 3.12.

Analyses proceeded in the following sequence: (1) baseline classification and comparison; (2) sub-stratification of the no-recovery pattern according to baseline LVEF and the direction of volumetric change; (3) sensitivity analyses using alternative structural and functional thresholds and LVEF measurement methods; and (4) exploratory analysis of deformation, myocardial work, and left atrial indices in relation to pattern membership and clinical outcome. Exploratory outcome analyses were considered hypothesis-generating.

### 2.6. Reproducibility Sub-Study

To address measurement reproducibility for the two threshold-defining variables, 15 patients were selected by simple random sampling from the 53-patient primary analytic cohort. For each patient, baseline and follow-up 3D-ESV and biplane 2D LVEF were re-measured: (1) by the original observer, blinded to the initial values (intraobserver repeatability), and (2) by a second, independent observer, blinded to both the initial values and the intraobserver repeat measurements (interobserver reproducibility). Agreement was quantified using the two-way random-effects, absolute-agreement intraclass correlation coefficient (ICC), Bland–Altman bias and 95% limits of agreement, and the coefficient of variation. We additionally determined, for each of these 15 patients, whether recovery-pattern assignment changed when the repeat or second-observer measurements were substituted for the original values.

## 3. Results

### 3.1. Baseline Characteristics of the Overall Cohort

Among the 101 enrolled patients, 58 completed the protocol follow-up visit at 12 ± 2 months; the remaining 43 patients did not, including one who died before the scheduled visit could take place, ascertained by telephone contact when the patient was called to schedule the appointment (Section 3.6). Among the 58 patients who completed the protocol follow-up visit, 53 had complete paired 3D-ESV and LVEF measurements and constituted the primary analytic cohort for the structural–functional recovery framework (participant flow summarized in Supplementary Figure S1). Infarct-related territory distribution is summarized in Table 1.

Patients completing the follow-up visit (*n* = 58) did not differ from those who did not (*n* = 43) in age, sex, baseline LVEF, or baseline GLS (all *p* > 0.05). Anterior/anterolateral territory was nominally less frequent among patients who completed the follow-up visit (18/58, 31.0%) than among those who did not (22/43, 51.2%; χ^2^ = 4.18, *p* = 0.041; Section 4.6). Five of the 58 patients with a completed follow-up visit were excluded from the primary analytic cohort because of incomplete paired imaging; these five were older and had lower baseline LVEF and GLS than the remaining 53. No formal statistical comparison was performed because of the small sample size.

Table 1 summarizes the available baseline characteristics of the 101 enrolled patients.

At follow-up, medical therapy for coronary syndrome, heart failure, secondary prevention, and comorbidities in the reassessed cohort (*n* = 58, available data) included antiplatelet therapy in 53/55 (96.4%), beta-blockers in 42/54 (77.8%), an ACE inhibitor or ARB in 29/55 (52.7%), an ARNI in 2/53 (3.8%), an SGLT2 inhibitor in 14/53 (26.4%), a mineralocorticoid receptor antagonist in 19/55 (34.5%), a statin in 55/55 (100%), and a diuretic in 8/55 (14.5%). These therapies were not compared across recovery patterns because of the small per-pattern cell sizes.

### 3.2. Paired Echocardiographic Changes from Baseline to Follow-Up

At reassessment, several systolic-performance measures improved at group level: 2D, AFI-derived, and 3D LVEF increased, while GLS became more negative. Global work index (GWI), global constructive work (GCW), and left atrial reservoir strain (LASR) also increased. Global wasted work (GWW) showed a smaller but FDR-significant increase; global work efficiency (GWE) did not change significantly. In contrast, neither 3D end-diastolic volume nor 3D-ESV showed a significant net change at the group level (Supplementary Table S2). This divergence between group-level functional improvement and heterogeneous volumetric trajectories motivated the patient-level cross-classification.

### 3.3. Structural–Functional Recovery Patterns

Cross-classification of the predefined structural and functional recovery criteria identified four structural–functional recovery patterns (Table 2, Figure 1A); the no-recovery pattern was the most frequent (27 patients, 50.9%). Overall, nearly one-third of patients (16/53, 30.2%) showed discordant recovery, improving along only one axis. Infarct territory (Section 2.1) was not associated with pattern membership (exact conditional 4 × 2 test, *p* = 0.851).

When analyzed as continuous, threshold-free variables across all 53 patients (Figure 1B), percent 3D-ESV reduction and ΔLVEF were moderately correlated (Spearman ρ = 0.353, bootstrap 95% CI 0.059–0.605, *p* = 0.0095).

Considered as two classifications of the same patients, the two criteria agreed only weakly: observed agreement was 69.8% and Cohen’s κ was 0.328 (bootstrap 95% CI 0.043–0.570). Disagreement was symmetrical, with seven patients meeting the structural criterion alone and nine meeting the functional criterion alone (exact McNemar *p* = 0.804), indicating that neither definition was systematically more permissive than the other. The discordance proportion was 30.2% (exact binomial 95% CI 18.3–44.3%).

Baseline LVEF differed nominally across the four recovery patterns (Kruskal–Wallis H = 8.56, *p* = 0.036), although this difference did not survive false-discovery-rate correction (FDR-*p* = 0.216). The no-recovery pattern had the highest baseline LVEF (50.6% vs. 45.7–47.0%), a finding likely influenced by mathematical coupling because functional recovery was defined using LVEF change (Section 4.6). Baseline GLS (*p* = 0.50) and GWI (*p* = 0.63) did not differ significantly across recovery patterns, nor did GCW or GWE (Supplementary Table S3). Baseline LASR was uniformly reduced versus normal reference values (~39% [18]) across recovery patterns, without between-pattern differences (21–25%, *p* = 0.90; Table 3).

### 3.4. Heterogeneity Within the No-Recovery Pattern

The 27 patients meeting neither criterion were not homogeneous. Eighteen (66.7%) had an increase in 3D-ESV, whereas nine (33.3%) showed stable or reduced 3D-ESV that remained below the 15% recovery threshold. Cross-classification by baseline LVEF (<50% vs. ≥50%) and 3D-ESV direction identified four configurations (Table 4): LVEF ≥ 50% with 3D-ESV increase (11/27, 40.7%), LVEF < 50% with deterioration (7/27, 25.9%), LVEF ≥ 50% with stable or sub-threshold reduction (6/27, 22.2%), and LVEF < 50% with stable or sub-threshold reduction (3/27, 11.1%). Given the post hoc design and cell sizes of three to eleven, this is descriptive heterogeneity, not a validated taxonomy.

### 3.5. Sensitivity Analyses of Recovery-Pattern Assignment

To assess robustness to the functional-recovery threshold, we repeated the analysis using a more conservative ≥10-point LVEF threshold [17]. Under this stricter threshold, the no-recovery pattern increased to 32 patients (60.4%), while concordant recovery and isolated functional recovery decreased to 5 (9.4%) and 4 patients (7.5%), respectively; isolated structural recovery increased to 12 patients (22.6%).

Varying the structural threshold from 15% to ≥10% or ≥20% reclassified 2 of 53 patients (3.8%) in each analysis.

Classification was more sensitive to the LVEF measurement method. Reclassification using 3D LVEF yielded concordant 10 [18.9%], isolated structural 7 [13.2%], isolated functional 13 [24.5%], and no recovery 23 [43.4%], with moderate agreement (62.3%; bootstrap 95% CI 49.1–75.5%; κ = 0.448; bootstrap 95% CI 0.247–0.633). AFI-derived LVEF (*n* = 52) yielded concordant 10, isolated structural 7, isolated functional 12, and no recovery 23, with moderate agreement (71.2%; bootstrap 95% CI 59.6–82.7%; κ = 0.578; bootstrap 95% CI 0.393–0.751; Supplementary Table S1). Overall, evidence for structural–functional discordance persisted, although individual pattern assignment varied with the LVEF measurement approach.

Three further analyses showed that discordance persisted across threshold- and modality-based sensitivity analyses. Across all 42 combinations of structural (≥5% to ≥30% 3D-ESV reduction) and functional (≥2 to ≥10 LVEF points) thresholds, discordance ranged from 20.8% to 52.8% and κ never exceeded 0.394 (Supplementary Table S4). When both criteria were derived from the same three-dimensional dataset—a ≥15% 3D-ESV reduction and a ≥5-point increase in 3D LVEF—discordance did not fall but rose to 37.7% (κ = 0.208; bootstrap 95% CI −0.060 to 0.479), and the AFI-derived comparison behaved similarly (36.5%; κ = 0.228; bootstrap 95% CI −0.044 to 0.495); these findings indicate that between-modality measurement differences are unlikely to be the sole explanation for the observed discordance. Replacing the absolute functional criterion with a relative one (≥10% relative LVEF increase) left the picture unchanged (discordance 32.1%; κ = 0.297; bootstrap 95% CI 0.018–0.545). Discordance was present in both baseline-LVEF strata and was greater among patients with baseline LVEF < 50% (41.4%; κ = 0.163) than among those with LVEF ≥ 50% (16.7%; κ = 0.500), the opposite of what a ceiling effect on the 5-point criterion would produce.

### 3.6. Exploratory Clinical Outcomes Across Recovery Patterns

One death, meeting the study’s major adverse cardiovascular event definition, occurred in a patient who did not complete the follow-up visit; it was ascertained by telephone contact when the patient was called to schedule the appointment, before any follow-up assessment could take place, and is not counted among events observed at the follow-up visit. Among the 58 patients who completed the follow-up visit, seven rehospitalizations, two implantable cardioverter-defibrillator implantations, and one cardiac resynchronization therapy-defibrillator implantation were recorded. At reassessment, 44 patients (75.9%) were in NYHA class I, 10 (17.2%) in class II, and 4 (6.9%) in class III. The death did not occur within the 53-patient analytic cohort, as this patient lacked complete paired imaging.

Clinical outcomes did not differ statistically across the four recovery patterns (Table 5). NYHA class ≥ II ranged from 0/9 in isolated functional recovery to 3/7 (42.9%) in isolated structural recovery (exact conditional 4 × 2 test, *p* = 0.140). When recovery patterns were collapsed according to the presence of functional recovery, NYHA class ≥ II occurred in 1/19 patients with functional recovery and 10/34 without it (Fisher’s exact OR = 0.13, *p* = 0.074); HF-related clinical-status and composite outcomes followed a directionally similar pattern. This association was imprecise and did not establish a stable clinical difference. In a descriptive post hoc comparison, clinical burden was highest among patients with both reduced baseline LVEF and volumetric deterioration, with the HF-related clinical-status outcome and NYHA ≥ II occurring in 6/7 (85.7%) and 5/7 (71.4%) patients, respectively. Event rates were lower overall in the remaining configurations, although estimates were unstable because of the very small subgroup sizes (exact test for NYHA ≥ II, *p* = 0.008). Given the small subgroup sizes, this comparison is hypothesis-generating, not confirmatory.

### 3.7. Exploratory Myocardial Mechanics and Left Atrial Function in Relation to Clinical Outcomes

Although baseline myocardial mechanics and LASR did not differentiate the four recovery patterns (Section 3.3), these markers showed numerical variation across the post hoc configurations within the no-recovery pattern (Section 3.4), with more favorable values among patients with preserved baseline LVEF (≥50%) than among those with reduced baseline LVEF (<50%) in the presence of volumetric deterioration (Supplementary Table S5).

GLS and myocardial work did not correlate with the continuous change in 3D-ESV or LVEF (all *p* > 0.25). In the exploratory marker–outcome screen, several baseline echocardiographic markers showed associations with clinical outcomes after false-discovery-rate correction (Supplementary Table S6): associations remained for GLS and several myocardial-work indices, as well as LASR. Given the small number of events and the correlated nature of these echocardiographic measures, these findings should be interpreted as exploratory rather than as evidence of independent prognostic effects. LASR, similarly unrelated to pattern membership, showed consistent associations across all three clinical outcomes predefined for the present analysis; because the composite outcome incorporates the HF-related clinical-status outcome, which itself incorporates NYHA class ≥ II, these should not be regarded as three independent replications of the same association.

Specifically, lower baseline LASR was associated with NYHA ≥ II, the HF-related clinical-status outcome, and the composite outcome in univariable analyses (Table 6). For NYHA ≥ II, each one-standard-deviation increase in LASR was associated with lower odds of the outcome (OR = 0.28, 95% CI 0.12–0.69, *p* = 0.005), with an apparent AUC of 0.776 (bootstrap 95% CI 0.589–0.931). Lower baseline LASR was similarly associated with the HF-related clinical status and composite outcomes, and these associations also remained below the false-discovery-rate threshold applied across the family of 21 marker-outcome combinations predefined for the present analysis. Given the small number of events (10–14), these estimates remain exploratory. Penalized Firth regression and nested-model sensitivity analyses yielded directionally consistent results (Supplementary Tables S7 and S8).

### 3.8. Measurement Reproducibility

In the 15-patient reproducibility sub-study, intraobserver agreement was excellent for both threshold-defining variables (ICC 0.969–0.997; coefficient of variation 1.2–3.1%; Supplementary Table S10), and no patient’s recovery-pattern assignment changed when intraobserver repeat measurements were substituted for the original values (0/15). Interobserver agreement was similarly excellent (ICC 0.945–0.998; coefficient of variation 2.1–2.8%), although recovery-pattern assignment changed for 1 of 15 patients (6.7%) when second-observer measurements were substituted; this patient’s follow-up LVEF change was within 2 percentage points of the ≥5-point functional-recovery threshold under both readings (3.0 and 5.0 points, respectively), illustrating the near-threshold instability discussed in Section 4.6.

## 4. Discussion

### 4.1. Structural–Functional Recovery as Complementary Axes

At the group level, systolic function measures improved after STEMI while 3D-ESV showed no consistent change (Section 3.2); considered alone, this pattern could be misread as an absence of structural recovery. The present findings show instead that this apparent group-level stability conceals substantial individual heterogeneity: structural and functional recovery capture complementary axes of post-infarction chamber response, with their cross-classification revealing patterns not apparent from either measure alone or from the group mean. Although significantly correlated (Spearman ρ = 0.353, *p* = 0.0095; Section 3.3), the two measures were only moderately related, with nearly one in three patients improving along only one axis. The literature-informed thresholds [15,16,17], predefined for the present analysis, are a practical tool, not the cause of the dissociation. Formal agreement analysis places this on a firmer footing: the two criteria agreed only weakly (κ = 0.328), disagreement was symmetrical, no combination of thresholds raised κ above 0.394, and discordance persisted—indeed increased—when both criteria were derived from a single three-dimensional acquisition, indicating that between-modality measurement differences are unlikely to be the sole explanation for the observed discordance (Section 3.5). This was not simply a threshold effect: even measured continuously, without any threshold, the relationship between 3D-ESV reduction and ΔLVEF was only moderate (Section 3.3). Single-parameter definitions, whether volume- or LVEF-based, reduce this process to a single metric, overlooking discordant recovery and obscuring meaningful heterogeneity in chamber response. Whereas Chimed et al. [10] classified post-infarction remodeling according to adverse structural change and the direction of LVEF change, our framework starts from the complementary question of recovery, defining structural reverse remodeling and an absolute increase in LVEF, predefined for the present analysis, from separate acquisitions. We note that LVEF is itself mathematically derived from end-diastolic and end-systolic volumes, so the two criteria are not physiologically independent; using separate imaging modalities reduces correlated measurement error but does not establish that discordant recovery reflects two biologically independent pathways. Adverse-remodeling classifications defined from an already-injured baseline do not distinguish stability from active recovery; conversely, our recovery-based classification cannot distinguish stability from deterioration among patients meeting neither recovery criterion. This heterogeneity is explored through the no-recovery-pattern sub-stratification (Section 3.4). A parallel shift is underway in cardiac MRI: Xiang et al. [19] used unsupervised clustering of biventricular and bi-atrial parameters to identify three data-driven post-STEMI phenotypes with incremental prognostic value beyond conventional volume-and-LVEF criteria, using a data-driven prognostic approach that differs from the threshold-based framework, predefined for the present analysis, used here.

Neither 3D-ESV nor LVEF is a pure measure of myocardial mechanical performance: both integrate myocardial function, geometry, and loading conditions, but not to the same degree, so their recovery may diverge.

Within the no-recovery pattern, most patients showed an increase in 3D-ESV, while others remained stable or showed reduction below the structural-recovery threshold (Section 3.4). This heterogeneity is examined further in Section 4.2.

### 4.2. The No-Recovery Pattern as a Heterogeneous Chamber Response

The no-recovery pattern is not a uniform group. Sub-stratification by baseline LVEF and 3D-ESV trajectory (Section 3.4) shows that absence of recovery encompasses markedly different structural and clinical patterns: among patients with preserved baseline LVEF, some showed a clear increase in 3D-ESV whereas others remained volumetrically stable; greater clinical burden was observed among those with reduced baseline LVEF and volumetric deterioration (Section 3.6). A binary classification collapses these patterns into a single category. This differs from previous “minimal-change remodeling” categories [9], which were defined by stability alone. Our no-recovery pattern requires absence of both structural and functional recovery, also including patients with reduced baseline LVEF but no volumetric increase (*n* = 3), a subgroup too small for meaningful analysis. This distinction is a hypothesis about the underlying remodeling state, not a demonstrated fact: with only one baseline and follow-up echocardiogram, it is not possible to determine whether this process has concluded or is evolving.

### 4.3. Chamber Recovery and Myocardial Mechanics

Baseline GLS and myocardial work did not differ across the four chamber-level recovery patterns and did not track continuous chamber change (Section 3.7), suggesting that baseline myocardial mechanical state was not closely aligned with subsequent chamber-level recovery in this cohort. This is physiologically plausible: strain and myocardial work characterize myocardial mechanical performance, whereas 3D-ESV and LVEF reflect the integrated chamber response, which also depends on ventricular geometry and loading conditions, not muscle function alone. Regional deformation may capture recovery-relevant information not evident in global indices: infarct-zone circumferential strain, for instance, has independently predicted post-STEMI functional recovery on CMR [20], suggesting that territory-specific mechanics warrant separate consideration from the global GLS and myocardial work indices used here. Yet several of these global mechanical measures were nonetheless associated with clinical outcomes in exploratory analyses (Section 3.7): markers that do not differentiate pattern membership may still capture clinically relevant aspects of myocardial function. The proposed framework provides context for interpreting myocardial mechanics, not a substitute for it.

### 4.4. Chamber Recovery, Left-Heart Integration, and Clinical Expression

Left atrial reservoir strain (LASR) adds complementary information by reflecting left-heart functional integration not captured by chamber-level recovery or myocardial mechanics alone. LASR itself did not differentiate the four recovery patterns, yet was associated with symptomatic and heart-failure outcomes in univariable models, consistent with prior post-STEMI reports [21]. These LASR associations remained below the false-discovery-rate threshold across the family of 21 exploratory comparisons predefined for the present analysis; several deformation and myocardial-work markers also showed exploratory FDR-significant associations with one or more outcomes (Supplementary Table S6). All such findings were derived from few events and require external validation. These markers are strongly intercorrelated, and their associations with clinical status attenuated after adjustment for baseline LVEF (Supplementary Table S6), indicating that they capture one axis of baseline myocardial impairment rather than providing demonstrated incremental prognostic value beyond LVEF. LASR also varied numerically across the post hoc no-recovery configurations, further suggesting that atrial function may capture information not reflected by chamber recovery alone. These measures therefore provide information different from LVEF: GLS and myocardial work assess myocardial mechanical performance, whereas LASR reflects the functional response of the left atrium to ventricular and loading conditions. This adds an atrial functional dimension to recent data-driven CMR phenotyping, which incorporated atrial size but not atrial function [19,22].

Together, these findings support the conceptual framework shown in Figure 2. Chamber recovery, myocardial mechanics, and atrial function provide complementary information, without implying a strict causal hierarchy among them. The exploratory differences observed within the no-recovery pattern require confirmation in larger cohorts.

### 4.5. Clinical and Research Implications

This classification provides a structured framework for describing chamber recovery beyond a single binary endpoint. The discordant patterns may reflect differences in myocardial recovery, loading, or ventricular geometry and should be tested with serial imaging in adequately powered external cohorts.

### 4.6. Limitations

This single-center, proof-of-concept study had a modest sample size, including only seven patients with isolated structural recovery, limiting statistical power and precluding adequately powered multivariable modeling. No a priori power calculation was performed; cohort size reflected consecutive enrollment. The finding that the no-recovery pattern had the highest baseline LVEF illustrates an inherent ceiling effect of threshold-based recovery definitions: patients with preserved baseline LVEF have less room to gain 5 points than those with depressed LVEF, a mathematical consequence of the threshold rather than a biological finding.

A single experienced observer performed all acquisition and analysis. Formal reproducibility was assessed post hoc for the two threshold-defining variables in a 15-patient random subset (Section 2.6 and Section 3.8): intraobserver and interobserver agreement were both excellent (ICC ≥ 0.94), yet recovery-pattern reclassification occurred in 0/15 (intraobserver) and 1/15 (interobserver) patients. This is illustrated by the only moderate agreement (κ = 0.448–0.578; Section 3.5) between biplane, 3D-, and AFI-derived LVEF, indicating that pattern assignment reflects measurement dependence rather than a limitation specific to any single method. Reproducibility for GLS and LASR was not assessed and remains a limitation. The reproducibility substudy assessed repeat analysis of the same stored image datasets and therefore addresses measurement/analysis reproducibility rather than acquisition-level reproducibility; individual pattern assignment may remain sensitive to measurement variability when values lie close to the predefined recovery thresholds.

The predefined thresholds were literature-based but not ideal for this population: the ≥10-point HFimpEF criterion was derived for chronic HFrEF populations with baseline LVEF ≤ 40%, whereas this cohort had higher LVEF (mean 48.6%), underscoring the importance of the threshold-free analysis in Section 3.3. Similarly, because baseline imaging was performed at 12–24 h post-PCI, before stunning and loading effects had fully resolved, the observed change at follow-up reflects both true remodeling and the later resolution of these early, transient effects. Follow-up was incomplete; attrition bias cannot be excluded, although reassessed and non-reassessed patients were broadly comparable for measured baseline characteristics (age, sex, LVEF, GLS; all *p* > 0.05). Anterior/anterolateral infarction was nominally less frequent among patients who completed the follow-up visit (χ^2^ = 4.18, *p* = 0.041), but this difference did not survive false-discovery-rate correction across the family of baseline comparisons (FDR-*p* = 0.204); this should not be taken as evidence against selection bias, particularly given that infarct territory is directly relevant to ventricular remodeling and recovery. A more directly relevant comparison—between the 53 patients constituting the primary analytic cohort and the 48 enrolled patients not included in it—likewise showed no significant differences in infarct territory, baseline 3D end-diastolic or end-systolic volume, time from symptom onset to PCI, pre-PCI thrombolysis, post-procedural TIMI 3 flow, age, sex, baseline LVEF, or baseline GLS (all *p* ≥ 0.08; Supplementary Table S9), although this comparison remains underpowered to exclude smaller effects.

Finally, the exploratory clinical-outcome analysis was markedly underpowered, with hard events too rare to analyze and cell sizes as small as *n* = 7–10 per recovery pattern. AUC estimates in Section 3.7 derive from as few as 10 events and should therefore be interpreted cautiously despite bootstrap optimism correction. Generalizability is further limited by the restriction to single-vessel coronary artery disease and by the relatively narrow, preserved baseline LVEF distribution of this cohort (mean 48.6%), which may not represent the broader contemporary STEMI population, including patients with multivessel disease or more severely reduced baseline LVEF.

## 5. Conclusions

Recovery after STEMI cannot be fully characterized by either volumetric or LVEF change alone, and failure to meet recovery criteria does not define a uniform trajectory. Discordance between structural and functional recovery persisted across alternative thresholds, LVEF measurement methods, and imaging modalities; however, assignment of individual patients to the four specific categories remained threshold- and measurement-dependent and should not be regarded as a fixed biological classification. Cross-classification of structural and functional recovery provides a chamber-level framework for recognizing this heterogeneity, while myocardial mechanics and LASR showed associations not captured by pattern membership in this exploratory cohort. Their exploratory associations with clinical outcomes warrant confirmation in larger cohorts. This framework integrates, rather than replaces, established measures and provides a basis for prospective validation of multiparametric post-STEMI recovery assessment.

## Figures and Tables

**Figure 1:**
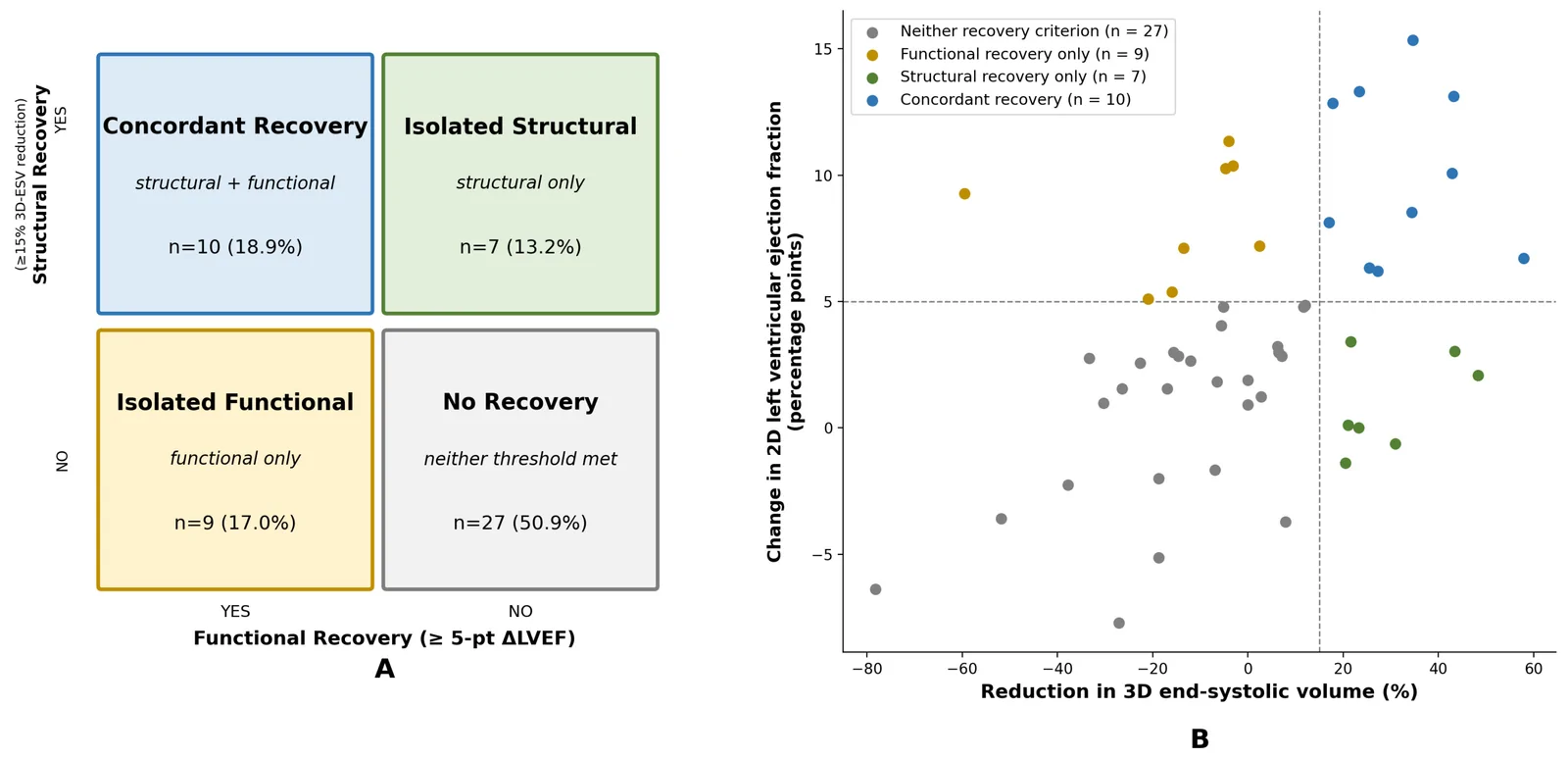
(**A**) Conceptual structural–functional framework: combining structural recovery (≥15% reduction in 3D end-systolic volume) and functional recovery (≥5-point absolute LVEF increase) identifies four structural–functional recovery patterns; values are *n* (%) for the primary analytic cohort (*n* = 53). (**B**) Continuous relationship between percent 3D-ESV reduction and ΔLVEF for the same 53 patients, colored by recovery pattern; dashed lines mark the primary thresholds.

**Figure 2:**
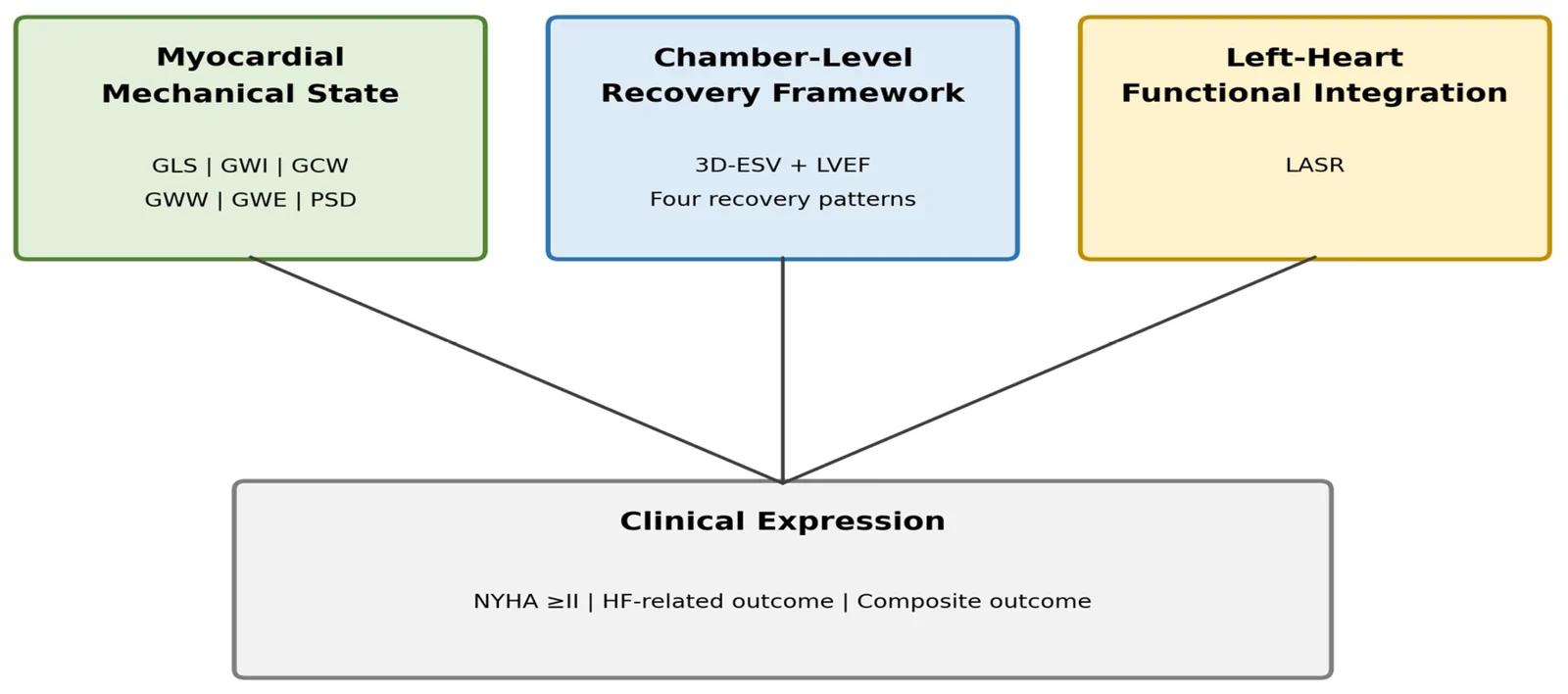
Conceptual framework of multiparametric post-infarction recovery assessment. Chamber-level recovery, defined by 3D-ESV and LVEF, identifies four structural–functional recovery patterns. Myocardial mechanical state and left-heart functional integration did not differentiate pattern membership, whereas selected baseline markers from these domains showed exploratory associations with clinical expression. The three domains are presented as complementary components of post-infarction assessment without implying a causal hierarchy. 3D-ESV, three-dimensional left ventricular end-systolic volume; LVEF, left ventricular ejection fraction; GLS, global longitudinal strain; GWI, global work index; GCW, global constructive work; GWW, global wasted work; GWE, global work efficiency; PSD, peak strain dispersion; LASR, left atrial reservoir strain; NYHA, New York Heart Association; HF, heart failure.

**Table 1:** Baseline demographic, clinical, angiographic, and echocardiographic characteristics of the overall study cohort (*n* = 101).

Characteristic	Value	*n*
Age, years (mean ± SD)	56.3 ± 10.6	101
Male sex, *n* (%)	68 (67.3%)	101
Hypertension, *n* (%)	53 (52.5%)	101
Diabetes mellitus, *n* (%)	21 (20.8%)	101
Current smoker, *n* (%)	68 (67.3%)	101
Body mass index, kg/m^2^ (median [IQR])	27.3 [25.0–31.1]	100
Infarct territory: anterior/anterolateral (LAD), *n* (%)	40 (39.6%)	101
Infarct territory: inferior ± posterior/lateral (RCA/LCx), *n* (%)	58 (57.4%)	101
Infarct territory: isolated lateral/posterior, *n* (%)	3 (3.0%)	101
Pre-PCI thrombolysis, *n* (%)	21 (20.8%)	101
Time from symptom onset to PCI, hours, median [IQR]	5 [3–7]	99
Post-procedural TIMI 3 flow, *n* (%)	92 (92.0%)	100
Baseline LVEF, % (mean ± SD)	48.6 ± 5.7	101
Baseline GLS, % (mean ± SD)	−13.6 ± 3.7	98
Baseline 3D end-diastolic volume, mL (median [IQR])	88.5 [75.5–106.8]	94
Baseline 3D end-systolic volume, mL (median [IQR])	46.5 [36.3–56.0]	94

Mean ± SD, median [IQR], or *n* (%). Percentages reflect available data. LVEF, left ventricular ejection fraction; GLS, global longitudinal strain; 3D, three-dimensional; LAD, left anterior descending artery; RCA, right coronary artery; LCx, left circumflex artery.

**Table 2:** Distribution of the four structural–functional recovery patterns and their corresponding changes in ventricular structure and function.

Recovery Pattern	*n* (%)	3D-ESV Reduction, %	Δ LVEF, Percentage Points	Δ GLS, Percentage Points
Concordant recovery	10 (18.9%)	32.4 ± 12.9	10.1 ± 3.4	−3.63 ± 4.02 (10 pairs)
Isolated structural recovery	7 (13.2%)	29.9 ± 11.6	1.0 ± 1.9	−2.98 ± 4.30 (6 pairs)
Isolated functional recovery	9 (17.0%)	−21.7 ± 27.4	8.8 ± 2.8	−3.92 ± 1.65 (9 pairs)
No-recovery pattern	27 (50.9%)	−13.9 ± 20.8	0.7 ± 3.5	−2.10 ± 3.44 (26 pairs)

Values are mean ± SD. Positive values in the 3D-ESV reduction column indicate a reduction in end-systolic volume (reverse remodeling), whereas negative values indicate an increase in 3D-ESV; for GLS, more negative values indicate improved systolic function. Smaller denominators for GLS reflect incomplete paired strain measurements.

**Table 3:** Baseline echocardiographic characteristics according to structural–functional recovery pattern.

Baseline Variable	Concordant	Isolated Struct.	Isolated Funct.	No Recovery	*p* (Kruskal–Wallis)
LVEF, %	46.46 [44.04–49.99]	46.97 [44.33–49.01]	45.65 [43.18–50.75]	50.62 [47.25–54.04]	0.036
GLS, %	−14.57 [−16.38 to −11.07]	−12.47 [−15.46 to −10.27]	−13.27 [−14.30 to −12.80]	−14.55 [−17.23 to −11.57]	0.50
GWI, mmHg%	1184 [870–1540]	1088 [868–1514]	968 [922–1027]	1085 [825–1392]	0.63
LASR, %	21 [17–24]	22 [16–33]	23 [21–24]	25 [17–28]	0.90

Values are median [IQR]. Group denominators were 10, 7, 9, and 27 for LVEF; 10, 6, 9, and 26 for GLS and GWI; and 10, 6, 9, and 27 for LASR. GCW and GWE showed no significant between-pattern differences and are reported in Supplementary Table S3. Kruskal–Wallis test; H denotes the test statistic. LVEF, left ventricular ejection fraction; GLS, global longitudinal strain; GWI, global work index; LASR, left atrial reservoir strain.

**Table 4:** Post hoc configurations among patients meeting neither structural nor functional recovery criterion (*n* = 27).

Configuration	*n* (%)	Baseline LVEF, %	LVEF Change, Points	3D-ESV Reduction, %
Baseline LVEF ≥ 50% with 3D-ESV increase	11 (40.7%)	53.7 ± 3.3	0.0 ± 3.0	−21.2 ± 14.0
Baseline LVEF ≥ 50% without 3D-ESV increase	6 (22.2%)	54.4 ± 4.1	1.8 ± 3.2	6.8 ± 4.8
Baseline LVEF < 50% with 3D-ESV increase	7 (25.9%)	45.2 ± 2.6	0.0 ± 5.0	−27.9 ± 24.4
Baseline LVEF < 50% without 3D-ESV increase	3 (11.1%)	47.0 ± 1.8	2.7 ± 0.7	4.5 ± 3.9

Values are mean ± standard deviation. Negative 3D-ESV reduction denotes an increase in end-systolic volume. The configurations were defined post hoc and were not subjected to confirmatory between-group inference. LVEF, left ventricular ejection fraction.

**Table 5:** Exploratory clinical outcomes according to structural–functional recovery pattern (*n* = 53).

Outcome	Concordant	Isolated Structural	Isolated Functional	No Recovery
NYHA class ≥ II	1/10 (10.0%)	3/7 (42.9%)	0/9 (0.0%)	7/27 (25.9%)
HF-related clinical-status outcome	2/10 (20.0%)	3/7 (42.9%)	1/9 (11.1%)	8/27 (29.6%)
Composite outcome	2/10 (20.0%)	3/7 (42.9%)	1/9 (11.1%)	9/27 (33.3%)

Values are events/available patients (%). Differences across recovery patterns were assessed using an exact conditional 4 × 2 test. *p*-values: NYHA class ≥ II, 0.140; HF-related clinical-status outcome, 0.490; composite outcome, 0.494. The HF-related clinical-status outcome is defined in Section 2.4. The composite outcome included the HF-related clinical-status outcome, rehospitalization, device implantation, major adverse cardiovascular events, or death. NYHA, New York Heart Association.

**Table 6:** Baseline left atrial reservoir strain as an exploratory univariable predictor of clinical status at reassessment.

Outcome	OR per 1-SD (95% CI)	*p*	Apparent AUC (95% CI)	Optimism-Corrected AUC	FDR *p*
NYHA class ≥ II (10/52 events)	0.28 (0.12–0.69)	0.005	0.776 (0.589–0.931)	0.779	0.019
HF-related clinical-status outcome (13/52 events)	0.29 (0.13–0.68)	0.004	0.775 (0.612–0.917)	0.775	0.019
Composite outcome (14/52 events)	0.36 (0.17–0.77)	0.009	0.730 (0.556–0.885)	0.728	0.023

Odds ratios were estimated using univariable logistic regression per one-standard-deviation increase in LASR. AUC confidence intervals used 10,000 stratified bootstrap samples; optimism correction used 2000 bootstrap refits. FDR p values were calculated using the Benjamini–Hochberg procedure across the family of 21 exploratory marker–outcome combinations. OR, odds ratio; CI, confidence interval; AUC, area under the receiver-operating-characteristic curve.

## Data Availability

The dataset analyzed in this study is available from the corresponding author upon reasonable request, subject to institutional and ethical approval.

## Supplementary Materials

The following supporting information can be downloaded at: https://www.mdpi.com/article/10.3390/biomedicines14102185/s1, Figure S1: Participant flow through enrollment, follow-up, and inclusion in the primary analytic cohort; Table S1: Recovery-pattern reclassification using 3D- and AFI-derived left ventricular ejection fraction; Table S2: Paired changes in principal echocardiographic measures between baseline and reassessment; Table S3: Baseline global constructive work and global work efficiency according to structural–functional recovery pattern; Table S4: Agreement between the structural and functional recovery criteria across 42 combinations of structural and functional thresholds (*n* = 53). Table S5: Baseline myocardial mechanics and left atrial reservoir strain across post hoc configurations of the no-recovery pattern (*n* = 27); Table S6: Univariable associations between baseline myocardial mechanics, left atrial reservoir strain, and clinical status at reassessment across the family of 21 marker–outcome combinations predefined for the present analysis; Table S7: Maximum-likelihood and penalized Firth logistic regression for baseline left atrial reservoir strain; Table S8: Nested-model analysis: contribution of baseline left atrial reservoir strain beyond functional recovery; Table S9: Baseline comparison between patients included in the primary analytic cohort and those enrolled but not included; Table S10: Intraobserver and interobserver reproducibility of 3D-ESV and biplane LVEF in a 15-patient random subset.
