# Supplementary material for: Beyond Remodeling: Four Structural–Functional Recovery Patterns as a Framework for Interpreting Ventricular Recovery After Reperfused STEMI
Source: Biomedicines. 2026 Sep 27;14(10):2185. doi: 10.3390/biomedicines14102185 (PMC13701567; doi:10.3390/biomedicines14102185)
Supplement: Supplementary file 1 [file biomedicines-14-02185-s001.zip › biomedicines-4562265-supplementary.pdf]

Supplementary Materials

Beyond Remodeling: Four Structural–Functional Recovery Patterns as a Framework for Interpreting Ventricular Recovery After Reperfused STEMI

Figure S1. Participant flow through enrollment, follow-up, and inclusion in the primary analytic cohort.

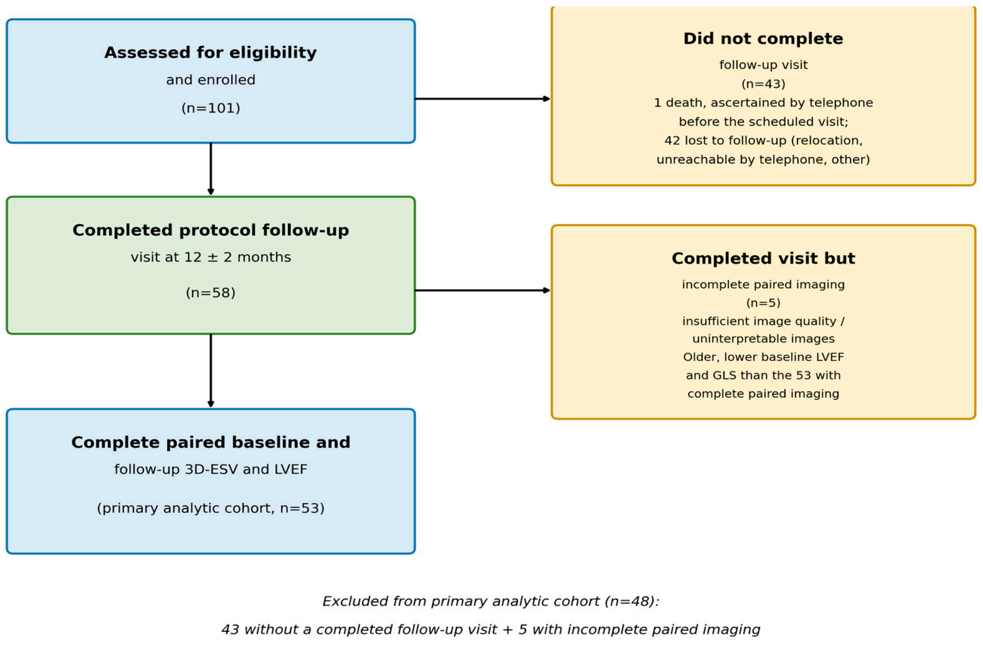

3D-ESV, three-dimensional end-systolic volume; LVEF, left ventricular ejection fraction; GLS, global longitudinal strain.

Table S1. Recovery-pattern reclassification using 3D- and AFI-derived left ventricular ejection fraction.

LVEF method	n	Concordant	Isolated structural	Isolated functional	No recovery	Agreement, % (95% CI)	κ (95% CI)	ρ with ΔLVEF (2D)
Biplane 2D LVEF (primary)	53	10	7	9	27	—	—	—
3D LVEF	53	10	7	13	23	62.3 (49.1–75.5)	0.448 (0.247–0.633)	0.442
AFI-derived LVEF	52	10	7	12	23	71.2 (59.6–82.7)	0.578 (0.393–0.751)	0.631

Recovery patterns were reassigned by replacing the biplane two-dimensional LVEF criterion with the stated method, keeping the structural criterion unchanged. Agreement is with the primary classification; confidence intervals are bootstrap percentile

intervals (2000 iterations). ΔLVEF differences of exactly 5.00 percentage points were treated as meeting the criterion. AFI, automated function imaging; κ, Cohen's kappa; ρ, Spearman correlation.

Table S2. Paired changes in principal echocardiographic measures between baseline and reassessment.

Measure	Pairs	Baseline	Reassessment	Median change	p	FDR p	r
2D LVEF, %	58	48.9 [44.4–51.6]	52.2 [49.1–56.8]	+3.0	<0.001	<0.001	0.67
AFI-derived LVEF, %	53	49.0 [46.0–52.0]	53.0 [50.0–56.0]	+4.0	<0.001	<0.001	0.68
3D LVEF, %	53	49.0 [46.0–52.0]	53.0 [51.0–56.0]	+4.0	<0.001	<0.001	0.67
GLS, %	52	-14.3 [-16.7 to -11.4]	-16.5 [-19.2 to -15.1]	-2.3	<0.001	<0.001	-0.80
GWI, mmHg%	52	1046.5 [824.0–1414.2]	1328.0 [1098.8–1526.2]	+179.0	0.003	0.005	0.47
GCW, mmHg%	52	1421.5 [1213.0–1826.2]	1833.0 [1531.0–2024.0]	+375.5	<0.001	<0.001	0.66
GWW, mmHg%	52	172.5 [108.2–212.2]	188.5 [134.8–268.8]	+28.0	0.020	0.030	0.37
GWE, %	52	88.0 [84.0–91.0]	89.5 [86.0–92.0]	+1.0	0.168	0.202	0.23
PSD, ms	52	58.0 [51.0–68.2]	56.5 [50.0–64.5]	-2.1	0.085	0.113	-0.28
LASR, %	54	22.0 [18.0–26.8]	26.5 [19.2–31.8]	+4.0	<0.001	0.002	0.53
3D end-diastolic volume, mL	53	91.0 [79.0–99.0]	85.0 [76.0–111.0]	+4.0	0.301	0.329	0.16
3D end-systolic volume, mL	53	47.0 [37.0–54.0]	41.0 [35.0–56.0]	+0.0	0.708	0.708	-0.06

Values are median [IQR]. Paired comparisons used the Wilcoxon signed-rank test; r is the matched-pairs rank-biserial effect size. FDR p values were obtained with the Benjamini–Hochberg procedure within this family of longitudinal comparisons. Global work efficiency was harmonized to percentage units at both time points before analysis. AFI, automated function imaging; GLS, global longitudinal strain; GWI, global work index; GCW, global constructive work; GWW, global wasted work; GWE, global work efficiency; PSD, peak strain dispersion; LASR, left atrial reservoir strain.

Table S3. Baseline global constructive work and global work efficiency according to structural–functional recovery pattern.

Baseline variable	Concordant (n=10)	Isolated structural (n=6)	Isolated functional (n=9)	No recovery (n=26)	H	p
GCW, mmHg%	1630.5 [1073.0–1911.5]	1373.5 [1277.5–1719.2]	1305.0 [1210.0–1421.0]	1489.5 [1229.5–1826.8]	1.84	0.606
GWE, %	89.0 [86.0–92.5]	84.5 [79.2–91.2]	88.0 [86.0–91.0]	88.0 [84.0–91.0]	1.55	0.670

Values are median [IQR]. H and p are from the Kruskal–Wallis test; these two variables belong to the family of six baseline echocardiographic comparisons predefined for the present analysis reported in Section 3.3. GCW, global constructive work; GWE, global work efficiency.

Table S4. Agreement between the structural and functional recovery criteria across 42 combinations of structural and functional thresholds (n=53).

Structural threshold	ΔLVEF ≥2 pts	ΔLVEF ≥3 pts	ΔLVEF ≥4 pts	ΔLVEF ≥5 pts	ΔLVEF ≥6 pts	ΔLVEF ≥8 pts	ΔLVEF ≥10 pts
3D-ESV reduction ≥5%	39.6 (0.236)	35.8 (0.281)	41.5 (0.155)	41.5 (0.138)	37.7 (0.208)	39.6 (0.146)	41.5 (0.090)
3D-ESV reduction ≥10%	43.4 (0.196)	32.1 (0.355)	34.0 (0.294)	34.0 (0.262)	30.2 (0.328)	32.1 (0.241)	34.0 (0.165)
3D-ESV reduction ≥15%	47.2 (0.144)	35.8 (0.278)	37.7 (0.208)	30.2 (0.328)	26.4 (0.394)	28.3 (0.296)	30.2 (0.209)
3D-ESV reduction ≥20%	50.9 (0.093)	39.6 (0.201)	41.5 (0.119)	34.0 (0.226)	30.2 (0.285)	32.1 (0.159)	30.2 (0.154)
3D-ESV reduction ≥25%	49.1 (0.166)	37.7 (0.236)	35.8 (0.219)	28.3 (0.313)	24.5 (0.368)	26.4 (0.199)	24.5 (0.167)
3D-ESV reduction ≥30%	52.8 (0.118)	41.5 (0.159)	39.6 (0.127)	32.1 (0.201)	28.3 (0.245)	22.6 (0.267)	20.8 (0.230)

Each cell gives the proportion of patients classified discordantly by the two criteria (%) and, in parentheses, Cohen's κ. Discordance ranged from 20.8% to 52.8% and κ never exceeded 0.394; the primary combination (≥15% 3D-ESV reduction and ≥5-point LVEF increase) is 30.2% and 0.328. 3D-ESV, three-dimensional end-systolic volume; LVEF, left ventricular ejection fraction; κ, Cohen's kappa.

Table S5. Baseline myocardial mechanics and left atrial reservoir strain across the post hoc configurations of the no-recovery pattern (n=27).

Marker	LVEF ≥50%, ESV increase (n=11)	LVEF ≥50%, no increase (n=6)	LVEF <50%, ESV increase (n=7)	LVEF <50%, no increase (n=3)	H	p
GLS, %	-17.1 [-20.1 to -15.7] (n=10)	-16.3 [-17.2 to -12.3] (n=6)	-11.7 [-12.5 to -11.2] (n=7)	-14.3 [-14.5 to -12.9] (n=3)	8.54	0.036
GWI, mmHg%	1157.0 [1019.8–1464.8] (n=10)	1391.5 [1136.2–1467.5] (n=6)	780.0 [670.5–954.0] (n=7)	1011.0 [914.5–1140.0] (n=3)	7.92	0.048
GCW, mmHg%	1604.0 [1349.2–1826.8] (n=10)	1801.0 [1509.8–2034.5] (n=6)	1081.0 [956.5–1338.5] (n=7)	1322.0 [1118.5–1671.0] (n=3)	8.26	0.041
GWE, %	90.0 [88.0–91.0] (n=10)	89.5 [88.2–92.2] (n=6)	84.0 [79.0–85.5] (n=7)	90.0 [85.5–90.5] (n=3)	9.26	0.026
PSD, ms	53.5 [47.1–61.5] (n=10)	62.5 [51.2–79.8] (n=6)	58.0 [56.0–91.0] (n=7)	50.6 [46.8–59.8] (n=3)	4.79	0.188
LASR, %	27.0 [22.5–30.0] (n=11)	17.5 [13.8–24.2] (n=6)	19.0 [12.0–24.0] (n=7)	25.0 [19.5–27.5] (n=3)	8.46	0.037

Values are median [IQR]; denominators are given per cell where they differ. H and p are from the Kruskal–Wallis test and are descriptive only: the configurations were defined post hoc, cell sizes range from three to eleven, and no correction for multiplicity was applied. GLS, global longitudinal strain; GWI, global work index; GCW, global constructive work; GWE, global work efficiency; PSD, peak strain dispersion; LASR, left atrial reservoir strain.

Table S6. Univariable associations between baseline myocardial mechanics, left atrial reservoir strain, and clinical status at reassessment across the family of 21 marker–outcome combinations predefined for the present analysis.

Marker	Outcome	Events/n	OR per 1-SD (95% CI)	p	FDR p
GLS	NYHA class ≥II	10/51	2.86 (1.22–6.67)	0.015	0.030
GLS	HF-related outcome	13/51	4.02 (1.58–10.20)	0.003	0.019
GLS	Composite outcome	14/51	3.33 (1.43–7.75)	0.005	0.019
GWI	NYHA class ≥II	10/51	0.35 (0.13–0.90)	0.030	0.041
GWI	HF-related outcome	13/51	0.34 (0.14–0.81)	0.015	0.030
GWI	Composite outcome	14/51	0.29 (0.11–0.72)	0.008	0.023
GCW	NYHA class ≥II	10/51	0.38 (0.15–0.92)	0.032	0.041
GCW	HF-related outcome	13/51	0.37 (0.17–0.84)	0.017	0.030

GCW	Composite outcome	14/51	0.38 (0.17–0.83)	0.015	0.030
GWW	NYHA class ≥II	10/51	1.42 (0.74–2.75)	0.294	0.294
GWW	HF-related outcome	13/51	1.77 (0.93–3.38)	0.084	0.093
GWW	Composite outcome	14/51	2.14 (1.08–4.25)	0.030	0.041
GWE	NYHA class ≥II	10/51	0.42 (0.20–0.89)	0.024	0.038
GWE	HF-related outcome	13/51	0.26 (0.11–0.64)	0.003	0.019
GWE	Composite outcome	14/51	0.24 (0.10–0.60)	0.002	0.019
PSD	NYHA class ≥II	10/51	1.85 (0.97–3.52)	0.062	0.072
PSD	HF-related outcome	13/51	1.85 (0.98–3.49)	0.058	0.072
PSD	Composite outcome	14/51	1.67 (0.91–3.09)	0.100	0.105
LASR	NYHA class ≥II	10/52	0.28 (0.12–0.69)	0.005	0.019
LASR	HF-related outcome	13/52	0.29 (0.13–0.68)	0.004	0.019
LASR	Composite outcome	14/52	0.36 (0.17–0.77)	0.009	0.023

Odds ratios are from univariable logistic regression per one-standard-deviation increase in the marker; for GLS, less negative values denote worse deformation. FDR p values were obtained with the Benjamini–Hochberg procedure across all 21 combinations. The markers are strongly intercorrelated (Spearman ρ up to 0.92 between GWI and GCW), and every association attenuates after adjustment for baseline LVEF; they should therefore be interpreted as one axis of baseline myocardial impairment rather than as independent predictors. OR, odds ratio; CI, confidence interval; FDR, false discovery rate.

Table S7. Maximum-likelihood and penalized Firth logistic regression for baseline left atrial reservoir strain.

**Outcome**	**Events/n**	**ML OR per 1-SD (95% CI)**	**Firth OR per 1-SD (profile 95% CI)**	**Wald p**	**Penalized LR p**
NYHA class ≥II	10/52	0.28 (0.12–0.69)	0.32 (0.12–0.67)	0.008	0.002
HF-related outcome	13/52	0.29 (0.13–0.68)	0.32 (0.13–0.66)	0.006	0.001
Composite outcome	14/52	0.36 (0.17–0.77)	0.39 (0.17–0.76)	0.012	0.004

Odds ratios are per one-standard-deviation increase in baseline LASR. Firth estimates use the penalized likelihood, with profile-likelihood confidence intervals. ML, maximum likelihood; LR, likelihood ratio.

Table S8. Nested-model analysis: contribution of baseline left atrial reservoir strain beyond functional recovery.

Outcome	Events/n	AUC, functional recovery only	AUC, + LASR	Optimism-corrected AUC, base	Optimism-corrected AUC, + LASR	LR p
NYHA class ≥II	10/52	0.664	0.875	0.663	0.856	<0.001
HF-related outcome	13/52	0.590	0.838	0.557	0.810	<0.001
Composite outcome	14/52	0.603	0.804	0.580	0.774	0.001

Models were fitted with penalized Firth logistic regression. Optimism correction used 2000 bootstrap refits. The likelihood-ratio p value compares the model containing functional recovery alone with the model additionally containing standardized baseline LASR. Given 10 to 14 events, these estimates are exploratory and internally validated only. AUC, area under the receiver-operating-characteristic curve; LR, likelihood ratio.

Table S9. Baseline comparison between patients included in the primary analytic cohort and those enrolled but not included.

Characteristic	Included (n=53)	Not included (n=48)	p
Anterior/anterolateral territory, n (%)	17/53 (32.1%)	23/48 (47.9%)	0.153
Baseline 3D-EDV, mL, median [IQR]	91.0 [79.0–99.0] (n=53)	85.0 [74.0–114.0] (n=41)	0.831
Baseline 3D-ESV, mL, median [IQR]	47.0 [37.0–54.0] (n=53)	44.0 [34.0–63.0] (n=41)	0.804
Time from symptom onset to PCI, h, median [IQR]	5.0 [3.0–6.0] (n=52)	5.0 [3.0–9.0] (n=47)	0.442
Pre-PCI thrombolysis, n (%)	15/53 (28.3%)	6/48 (12.5%)	0.084
Post-procedural TIMI 3 flow, n (%)	47/52 (90.4%)	45/48 (93.8%)	0.717
Age, years, mean ± SD	56.0 ± 9.7	56.8 ± 11.5	0.691
Male sex, n (%)	36/53 (67.9%)	32/48 (66.7%)	1.000
Baseline LVEF, %, mean ± SD	48.7 ± 5.3	48.6 ± 6.1	0.886
Baseline GLS, %, mean ± SD	-14.0 ± 3.6 (n=51)	-13.3 ± 3.9 (n=47)	0.246

Comparisons used the Mann-Whitney U test for continuous variables and Fisher's exact test for categorical variables. 'Included' denotes the 53 patients with complete paired baseline and follow-up 3D end-systolic volume and biplane 2D LVEF constituting

the primary analytic cohort; 'Not included' comprises the remaining 48 enrolled patients (43 without a completed follow-up visit and 5 with a completed visit but incomplete paired imaging). Denominators are given for continuous variables with missing data (baseline 3D-EDV, 3D-ESV, time from symptom onset to PCI, and GLS); all other variables were available for the full group indicated in the column heading. Given the modest sample size, the absence of a statistically significant difference for anterior/anterolateral territory and pre-PCI thrombolysis does not establish equivalence between the two groups, and a clinically meaningful imbalance in these variables cannot be excluded. 3D-EDV, three-dimensional end-diastolic volume; 3D-ESV, three-dimensional end-systolic volume; PCI, percutaneous coronary intervention; TIMI, Thrombolysis in Myocardial Infarction; LVEF, left ventricular ejection fraction; GLS, global longitudinal strain.

Table S10. Intraobserver and interobserver reproducibility of 3D-ESV and biplane LVEF in a 15-patient random subset.

Variable	Comparison	ICC (95% CI)	Bias	95% Limits of Agreement	CV, %
Baseline 3D-ESV, mL	Intraobserver	0.993 (0.98–1.00)	0.87 mL	−3.81 to 5.54	3.1
Follow-up 3D-ESV, mL	Intraobserver	0.997 (0.99–1.00)	1.20 mL	−2.21 to 4.61	2.3
Baseline LVEF, %	Intraobserver	0.969 (0.91–0.99)	0.00%	−2.57 to 2.57	1.9
Follow-up LVEF, %	Intraobserver	0.987 (0.96–1.00)	0.17%	−1.63 to 1.98	1.2
Baseline 3D-ESV, mL	Interobserver	0.992 (0.96–1.00)	1.60 mL	−2.64 to 5.84	2.8
Follow-up 3D-ESV, mL	Interobserver	0.998 (0.99–1.00)	0.60 mL	−3.01 to 4.21	2.5
Baseline LVEF, %	Interobserver	0.963 (0.89–0.99)	−0.13%	−2.99 to 2.72	2.1
Follow-up LVEF, %	Interobserver	0.945 (0.82–0.98)	−0.89%	−4.17 to 2.38	2.2

ICC, two-way random-effects, absolute-agreement intraclass correlation coefficient, single measures. Bias and limits of agreement from Bland-Altman analysis (second reading minus original). CV, coefficient of variation. Fifteen patients were selected by simple random sampling from the 53-patient primary analytic cohort. Recovery-pattern reclassification (relative to the original assignment) occurred in 0/15 patients (0%) using intraobserver repeat measurements and 1/15 patients (6.7%) using interobserver measurements; the reclassified patient's follow-up LVEF change was 3.0 percentage points under the original reading and 5.0 percentage points under the second observer's reading, spanning the ≥5-point functional-recovery threshold. 3D-ESV, three-dimensional end-systolic volume; LVEF, left ventricular ejection fraction.
